# Community awareness and cultural beliefs on female genital mutilation in Ibadan, Oyo State: Insights from a localised intervention

**DOI:** 10.1186/s12905-026-04483-2

**Published:** 2026-04-27

**Authors:** Semiyu Gbadebo Hammed, Enemona Jacob

**Affiliations:** School of Health, Medicine and Life Sciences, College Lane, University of Hertfordshire, Hatfield, Hertfordshire AL10 9AB UK

**Keywords:** Female genital mutilation, Young women, Narrative inquiry, Qualitative research, Reproductive effects, Awareness programmes

## Abstract

**Background:**

Female Genital Mutilation (FGM) remains a significant public health issue in Nigeria, particularly in Ibadan, with a prevalence rate of 38%. Despite increased awareness and advocacy, FGM persists as a rite of passage, chastity, or cultural custom.

**Objective:**

To investigate the impact of community awareness programs and cultural beliefs regarding FGM in Ibadan Oyo State, examining traditional misconceptions, cultural justifications, and the impact of awareness programs.

**Method:**

This is a qualitative study among female young adults of reproductive age (18–49 years), who participated in semistructured in-depth interviews using a narrative inquiry qualitative research approach. Insights into participants’ experiences, perspectives, and attitudes impacted by awareness programs, as well as the interaction of cultural phenomena with public health education were revealed through thematic analysis of the data.

**Results:**

Participants perceived that awareness programs and education have reduced FGM prevalence by providing accurate information, challenging misconceptions, and offering alternatives. Schools and community-based initiatives empower younger generations, but bridging generational knowledge gaps remains a challenge.

**Conclusion:**

Eliminating FGM in Ibadan requires not only disseminating information but also sustaining culturally respectful, context-specific advocacy. Long-term progress lies in embedding anti-FGM education in everyday institutions and empowering future generations to advocate for change.

**Supplementary Information:**

The online version contains supplementary material available at 10.1186/s12905-026-04483-2.

## Background

Female genital mutilation (FGM) is a cultural practice that involves the partial or complete removal of female external genitalia for nonmedical reasons, often driven by religious and societal beliefs [32, 33, 47, 49, 58, 76]. Despite being a global human rights violation, FGM continues to be practiced in various nations, including Nigeria, where it is widespread and has varying prevalence rates across different areas [25, 27, 45, 64, 66]. Female genital mutilation, a widespread practice in Africa, Asia, and the Middle East, is deeply rooted in sociocultural factors and influenced by social norms and beliefs [11, 18, 34, 68].

FGM in West Africa, which has historical roots beyond colonial times [30, 41, 71], varies by regional practices across ethnic and religious groups, rationalizing the act of maintaining chastity, improving marriage opportunities, or adhering to community cultural norms [4, 9, 53]. Nigeria, with high prevalence rates of female genital mutilation, is a culturally significant practice among various ethnic groups, including the Yoruba, Igbo, and Hausa communities [14, 51, 60]. The method is often seen as a cultural imperative, with variations in its manifestation and intensity across different locations [25, 57].

Nigeria has the highest absolute number of FGM cases in Africa, with a prevalence rate of approximately 25% among women of childbearing age [72]. The practice continues across all major ethnic groups in Nigeria, with some areas indicating prevalence rates reaching 38% [16, 86]. Despite heightened awareness, the incidence of FGM has varied among Nigeria’s six geopolitical zones over the last twenty years [14, 61]. A study by Gwarzo [36] in one of the Northwestern part of Nigeria indicated that while 93.6% of mothers were cognisant of FGM, 67.2% who had experienced the procedure perpetuated the practice with their daughters, and only 10.4% acknowledged its detrimental effects. Oyo State, located in southwestern Nigeria, is home to the Yoruba people, one of the largest ethnic groups in Nigeria [6, 88]. The practice of FGM in Oyo State, as in many other parts of Nigeria, is deeply embedded in cultural and traditional practices. In Yoruba culture, FGM has been practiced for centuries as a way to preserve virginity, control women’s sexuality, and ensure successful marriage [3, 66, 70]. It is often seen as an important rite of passage that marks a girl’s transition into womanhood. However, Oyo State is leading efforts to combat FGM in southwestern Nigeria, with NGOs, health organizations, and the Nigerian government promoting alternative rites of passage [2, 38, 67].

In urban areas such as Ibadan, the state capital, awareness of the dangers of FGM has led to a decrease in its prevalence [69, 86]. However, rural communities in Oyo State face challenges in eliminating FGM due to cultural pressure [40], and the government collaborates with organizations to enforce legislation, educate, and support community-led efforts [40, 55, 7]. This article explores the community awareness and cultural beliefs surrounding FGM in Ibadan, Oyo State, Nigeria. It analyses the prevailing knowledge levels, misconceptions, cultural justifications, and influence of traditional norms on the continuation of the practice.

### Theoretical framework

To support this study, the socio-ecological model (SEM) originally proposed by Bronfenbrenner in 1979 was adopted [77]. The SEM offers a comprehensive framework for understanding the multiple influences on FGM practices and the effects of awareness programs in Ibadan, Nigeria. In the context of FGM, the SEM provides a detailed approach to examining how awareness campaigns influence young women’s knowledge, attitudes, and behaviours at various levels, including individual, interpersonal, community, organizational, and policy levels [77]. This approach enables the design of interventions that create synergies between individual and social-environmental factors (as described in the SEM), potentially leading to more effective and sustainable behaviour-change strategies in the fight against FGM [91].

## Methods

### Study design

The study utilised narrative inquiry as a qualitative research framework to investigate participants’ experiences of FGM and associated awareness programmes in Ibadan, Nigeria. This method provided nuanced insights into complex topics such as FGM [23, 46], which took the perspectives of participants into account.

The study focused on personal stories and how cultural customs and awareness initiatives influenced behaviour and decision-making. Ibadan is a diverse society with strong roots in cultural practices such as FGM, making it an ideal location for awareness programs aimed at positive change [12, 5].

The research philosophy is social constructionism, which holds that reality is socially constructed rather than based on statistical inference. This meant that the findings of this research are based on the researchers’ interpretations of participants’ interview responses. This means that the findings are subjectively constructed. Secondly, the principal researcher has lived in Ibadan, Nigeria, before, and awareness of the FGM practice and a dislike for it may have led to an overemphasis on its negative consequences. However, the participants’ words were directly used to explain the findings and discussions. This has also been stated succinctly and transparently, how the study was carried out.

### Study location

The study was conducted in Ibadan, Oyo State, Nigeria, the capital and third-largest city in the country, with a population of over 2,649,000, as of 2021 [5]. It is known for its culture, education, and historical significance on the prevalence of FGM, as well as ongoing initiatives to influence this African culture, thus making it an ideal location for awareness programs on the FGM issue. Yoruba is the primary ethnic group in Ibadan, and the city has 11 local government areas (LGAs) covering the metropolitan area. This heterogeneity enabled a diverse population and improved understanding of FGM practices and awareness levels [12]. This research aims to contribute to positive changes in the community.

### Study participants: inclusion/exclusion criteria

This study focused on young and older females between 18 and 49 years who primarily reside within Ibadan city, Nigeria, and who have either experienced FGM or participated in awareness campaigns regarding female genital mutilation (FGM). The population is selected for its dual significance as both a target group for FGM interventions and a potential community change agent. Those who did not meet all these criteria were excluded from participating in the study.

### Sampling and sample size

The study used purposive and snowball sampling techniques to select participants relevant to its objectives, with a focus on women with FGM-related experiences [15, 19, 52]. Snowball sampling allows for additional participants through referrals, broadening the scope while maintaining relevance [74].

The sample size of 22 participants out of the 44 people who expressed interest in the study interview ranged from 18 to 49 years of age. The study stopped interviewing after the 22nd participant when data saturation was reached. Data saturation, or “information redundancy,” is a widely accepted threshold for determining a sufficient sample size in thematic and narrative studies [26]. They were selected across 7 local governments in Ibadan and educated to university level, which helped to ensure thoroughness while balancing methodological thoroughness with practical constraints [85, 92]. 13 of them were between 18 and 35 years, while 9 were between 36 and 49 years; 5 students and 9 were self-employed, while 8 were employed in different sectors. (Table 1).

**Table 1:** Participants’ demographic representation is shown in the table below

S/*N*	Age	Region	Education Level	Marital Status	Employment Status
1.	40	Ibadan SW	Tertiary	Married	Self-employed
2.	20	Oluyole South	Tertiary	Single	Student
3.	42	Ibadan SE	Tertiary	Married	Self-employed
4.	43	Egbeda	Postgraduate	Married	Self-employed
5.	44	Akinyele	Tertiary	Married	Self-employed
6.	29	Ona Ara	Postgraduate	Married	Employed
7.	40	Egbeda	Postgraduate	Married	Employed
8.	24	Akinyele	Tertiary	Single	Student
9.	30	Egbeda	Postgraduate	Married	Self-employed
10.	34	Akinyele	Tertiary	Married	Self-employed
11.	38	Egbeda	Tertiary	Married	Self-employed
12.	44	Ibadan SE	Postgraduate	Married	Employed
13.	28	Egbeda	Tertiary	Married	Employed
14.	27	Akinyele	Postgraduate	Married	Self-employed
15.	41	Egbeda	Postgraduate	Married	Student
16.	28	Akinyele	Tertiary	Single	Self-employed
17.	30	Akinyele	Tertiary	Married	Employed
18.	35	Akinyele	Tertiary	Married	Employed
19.	24	Akinyele	Postgraduate	Single	Student
20.	39	Egbeda	Tertiary	Married	Employed
21.	31	Ido	Postgraduate	Married	Employed
22.	31	Ibadan SW	Postgraduate	Single	Self-employed

### Recruitment, consent processes, and interview procedures

Participant recruitment was conducted through various online platforms, including WhatsApp and Telegram. Participants were provided with informational posters and invitation messages, and digital consent was obtained before interviews were scheduled via Microsoft Teams to streamline and ensure ethical recruitment. Through Qualtrics, participants accessed a participant information sheet and consent form, which provided details on the study’s purpose, voluntary participation, confidentiality assurance, and withdrawal procedures.

The study location, Ibadan, Oyo state, is a high-education profile state in Nigeria. This means that the literacy level is very high, and the target population aged 18–49 belongs to a highly educated generation(s) who were born and raised with technology [29]. They are a combination of the millennial generation and Gen Z, both of whom are known to be highly educated and familiar with digital channels [29]. As Oyo State is typical of a high level of education, and the participants are from the Y and Z generations, they were deemed educated and thus suitable to participate in the online interview for the study.

The research used online semistructured interviews for data collection because of their adaptability and comprehensiveness, making them ideal for exploring sensitive issues such as female genital mutilation (FGM) and awareness programs. These interviews allowed participants to articulate their thoughts and provide a rich narrative that aligns with the principles of qualitative research.

The interview guide used in this study was developed for this study and is included in the supplementary materials. The interview guide was designed to ensure clarity and cultural sensitivity and to encourage participants to share their experiences in a safe environment. The questions included “What are the main reasons for FGM practices in your community?” “What role do you think awareness programs can play in the prevalence of FGM?” “How do you feel about awareness programs having any impact on the practice of FGM in your community?” and “Have you witnessed changes in attitudes due to awareness programs?” These questions enabled an in-depth understanding and data collection on awareness programs. The interviews were held via Microsoft Teams, allowing for personal time and lasting 45–60 min.

The study utilised a semi-structured format to facilitate lively discussions and maintain consistency in participants’ responses in line with the research objectives. Iterative refinement of the interview questions and best practices for qualitative research improved the consistency and dependability of the research process and its findings.

The interview was conducted in English, and participants’ responses were recorded. This method upheld ethical standards and ensured that participants’ voices were central to the study’s results, which examined the interactions among cultural norms, personal experiences, and the impact of FGM awareness campaigns.

### Ethical approval

The University of Hertfordshire Health, Science, Engineering, and Technology Ethics Committee (Protocol number: cLMS/PGT/UH/05727; dated August 12, 2024) approved the study, adhering to strict protocols. The participants were informed about this, and they signed consent through a secure online platform (Qualtrics), and personal identifiers were replaced with anonymized codes.

### Data management and analysis

The research adhered to ethical legal provisions and maintained robust data management and storage systems [79]. Personal information and recordings were kept only for research purposes, and after each was converted into a transcript, they were securely destroyed. Transcripts and recordings underwent specific anonymization actions to prevent recognizable data from being recovered [87]. The backup files were stored in a password-protected OneDrive account at the University of Hertfordshire, which helped minimise unauthorized access and potential data breaches. This ensured participant confidentiality and data integrity.

The study ensured data integrity through encryption and strict access protocols, allowing only the main investigator to handle the data [31]. The Participant Information Sheet explained the secure storage, processing, and erasure of information, fostering trust among participants [42]. Backup activities during data retention were sufficient to protect against loss and corruption. After completion, electronic files were permanently deleted, and physical copies were shredded, ensuring compliance with the General Data Protection Regulation (GDPR) and institutional policies [42].

The study utilized thematic analysis to analyse qualitative data, focusing on complex, context-specific issues such as FGM and awareness programs. This method allows for a comprehensive examination of cultures, individuals, and groups, making it an effective tool for understanding society’s relationship with its own stories [21, 73, 75]. The study followed Braun and Clarke’s six-phase process for data analysis (1) familiarisation with data, (2) generating initial codes, (3) searching for themes, (4) reviewing themes, (5) defining and naming themes, and (6) writing the report [21, 20]. The first step involved familiarising with the data by reviewing Microsoft Teams transcripts and saving them to the University of Hertfordshire’s secure OneDrive. This stage allows the researcher to delve thoroughly into the data and gain bias-free preliminary knowledge of the content [28, 37]. The next step involved coding the data and systematically labelling related ideas and patterns [56, 84]. The researcher used manual coding to gain a personal connection to the data and better recognize subtle insights. The codes were combined in clusters to form initial themes, focusing on participants’ attitudes towards FGM and their associations with effective awareness campaigns [90, 22, 93]. The themes were reviewed iteratively to ensure coherence and relevance to the research questions. This process involved checking the alignment of themes with the coded data and refining them to address any overlap or inconsistencies. These themes were then defined and named based on their scope and significance, ensuring they accurately convey the key insights from the data. The final step involved synthesizing the themes into a cohesive narrative. This phase included integrating direct participant quotes to illustrate the themes and ensure the findings were grounded in the data. The report was structured to address the research objectives, linking themes to the broader cultural and social context of FGM.

## Results

A total of 44 individuals aged 18**–**49 years expressed interest in participating in the study, while only 22 participants (50%) participated in the interview conducted via Microsoft Teams (12 graduate and 10 postgraduate individuals). Recruitment was conducted across 7 local government (LG) areas within the Ibadan metropolis (Akinyele LG, Egebda LG, Ido LG, Ibadan SW LG, Ibadan SE LG, Oluyole South LG, and Ona Ara LG). The thematic analysis identified four central themes: shifting beliefs and behaviour change, resistance to cultural norms, family stability, and the culture of volunteering. (Table 2).

**Table 2:** The codes and themes generated from participants responses are shown in the table below

Codes	Themes
- Not circumcising daughters (1,4,5,10,11,16,20,22)	Shifting Beliefs and Behavior
- Reduction in FGM practice (2,3,5,6,7,17,20,21,22)	Change
- Discouraging it among others (1,2,5,7,9,13,15,16,18,20,21)	
- Change in community attitude (3,5,7,14,18,21,22)	
- Change wrong traditional belief (4,5,6,8,15,19,21)	Counterculture
- Does not control a child and keep family honour (1,4,10,13,21)	
- Enlighten mothers who think FGM protects (8,9,11,12,13,19,21)	
- Low libido in marriage, open up FGM issue to spouse (10,22)	Family Stability
- Religious and community leaders should help spread awareness (10,12,16,18,21)	Culture of Volunteering
**-** Volunteer in community/school awareness programs (9,12,17,21)	

### Shifting beliefs and behavior change

Shifting beliefs and behavior change are central to the effectiveness of awareness programs aimed at eradicating Female Genital Mutilation (FGM). Participants perceived that awareness programs were effective in influencing individual beliefs about FGM, leading to gradual behavioral changes. The participants highlighted how exposure to school-based and community awareness initiatives equipped them with knowledge about the physical and psychological risks associated with FGM, prompting a shift in perspectives.

For example, a participant shared, “After attending awareness programs, I decided not to circumcise my daughters” (p11). This quote emphasizes how exposure to information through educational campaigns can empower individuals to make informed decisions, thereby breaking generational cycles of FGM.

Furthermore, the participants noted that schools play a significant role in spreading anti-FGM messages to young people, who can then influence their families and broader communities. Another participant said:

“Weekly sessions in schools teach girls about harmful traditional practices” (p17).

This shows that school-based programs educate girls about FGM risks and consequences, empowering the next generation and challenging cultural norms. Schools provide safe spaces for girls to learn without fear of judgment, fostering new perspectives.

The participants also stated that awareness campaigns held in clinics and public health centers are highly effective. Additionally, one of the participants noted:

“Awareness has helped reduce the practice. It’s more common in cities than in rural areas” (p5).

This indicates that where awareness initiatives are more prevalent, there tends to be a noticeable reduction in FGM practices.

These responses show that young adults, particularly in urban areas, have begun rejecting FGM due to an improved understanding of its consequences. While individual participants report significant changes in their understanding and decisions, the transition to collective behavioural change is more gradual because of persistent cultural pressures.

### Counterculture

Participants disclosed that awareness campaigns have empowered individuals to question the deeply rooted cultural norms that perpetuate female genital mutilation (FGM). These norms, often tied to family honour, morality, and social conformity, present significant barriers to change. Many participants highlighted that FGM persists because of unchallenged traditions. For instance, a participant stated:

“Some families still enforce it without questioning the tradition” (p13).

A participant who is also a schoolteacher remarked:

“People are not fully informed on why it’s done; they just follow tradition” (p15).

Awareness programs challenge this ignorance, enabling individuals to critically evaluate these practices. Despite individual belief changes, translating this into broader collective action remains difficult. As one of the younger participants noted,

“Older people say it helps control a child and keeps the family honour” (p1).

This statement reflects how society pressures younger generations when rejecting FGM. Similarly, one of the participants noted that:

“Some mothers still enforce circumcision because they believe it’s their duty” (p13).

A significant barrier to progress is the generational divide in attitudes toward FGM. Younger individuals exposed to awareness programs often reject FGM, whereas elderly individuals view it as a cultural necessity. Another participant, who is also a schoolteacher, shared the following:

“In some families, the elders decide everything, and they see FGM as something that must be done” (p5).

This tension complicates efforts to eradicate the practice. The participants emphasized the importance of social support in resisting cultural norms. A participant who is a school teacher noted:

“Involving community leaders helps spread awareness and reduce resistance” (p12).

By leveraging trusted figures, awareness campaigns can foster collective shifts in attitudes, reducing the stigma of abandoning FGM.

### Family stability

Findings from participants that awareness programs not only target individuals but also emphasize the role of families and communities in creating a supportive environment for rejecting FGM. The participants emphasized how family stability fosters open communication, enabling younger members to voice their opposition to FGM without fear of alienation.

“I have a problem with it in my marriage, but I only find ways around it. Then, I open up to my spouse. You get it? I open up to my spouse, so this brings a kind of peace to my home” (p10). From this participant’s response, she demonstrated how low libido and a lack of sexual desire during sexual intercourse have affected her and almost caused problems in her marriage. After she opened up to her spouse about what she had been going through, they were able to tackle the issue together, which has helped to save her marriage from marital instability.

### Culture of volunteering

A culture of volunteering within communities was also seen as enhancing the reach and impact of awareness programs. Participants shared that individuals who had benefited from these campaigns often volunteered to spread anti-FGM messages, creating a ripple effect that influenced others. A participant who is an awareness volunteer declared the following:

“Realizing it is harmful, awareness has helped me discourage it among others” (p15).

One of the older participants noted:

“Religious and community leaders should help spread awareness” (p12).

These findings suggest that collective volunteer action and leadership drive community-wide advocacy against FGM. By encouraging families to work together and promoting volunteer-driven advocacy, awareness programs leverage familial and communal bonds to support the abandonment of FGM

## Discussion

This study investigated community awareness and cultural beliefs about Female Genital Mutilation (FGM) in Ibadan, Oyo State, using insights from a localized intervention. Using Urie Bronfenbrenner’s Socio-Ecological Model (SEM), the findings show that awareness initiatives shape perceptions across multiple ecological levels, while also highlighting the limitations of awareness alone in challenging deeply rooted cultural norms.

Studies have revealed that awareness programs have played a significant role in challenging traditional beliefs and reshaping behaviours related to FGM [35, 82]. Participants perceived that awareness programs are particularly effective in urban areas, where schools and public health campaigns provide structured opportunities for education, because they help change people’s perceptions. Awareness campaigns often focus on the physical and psychological consequences of FGM [8, 63], such as its health risks and impact on mental well-being [44]. These messages are tailored to dispel myths, including the belief that FGM is necessary for preserving chastity or preventing promiscuity.

Several barriers have been shown to limit the effectiveness of awareness programs in combating FGM, including countercultural beliefs and geographic disparities [14, 55]. Among these, cultural resistance emerged as the most significant challenge [35, 82]. FGM is deeply entrenched in many communities, where it is viewed as a rite of passage, a symbol of purity, and a means of controlling female sexuality [4, 9]. These cultural justifications create strong social pressure to conform, making it difficult for individuals to reject the practice even when they are aware of its risks.

The success of awareness programs in changing beliefs lies in their ability to foster critical thinking [46, 83] among participants, particularly young adults. Initiatives that educate women about health risks and legal prohibitions of FGM empower them to make informed decisions, aligning with the perspectives of Njue et al. [59] and Odera et al. [65].

In addition, younger generations exposed to these programs often act as agents of change within their families, introducing alternative perspectives that challenge entrenched cultural norms. However, in a similar observation reported by Abdulnor [1], while individual beliefs may change, behavioral shifts at the community level are often slower due to persistent social pressures and deeply rooted traditions.

The transfer of knowledge about the dangers of FGM to younger generations is one of the most effective strategies for reducing its prevalence [48, 54]. Schools play a pivotal role in this process [50, 55], serving as safe spaces where adolescents can learn about FGM without fear of judgment [89]. Awareness campaigns targeting young people often include interactive sessions, open discussions, and educational materials that emphasize the medical and legal implications of the practice [10, 24, 43]. These efforts not only inform young adults but also create a ripple effect, as informed individuals share their knowledge with peers and family members.

Furthermore, awareness programs should adopt a multi-generational approach that includes parents, caregivers, and community leaders alongside adolescents [78]. Families are integral to community structure, providing social ties and support systems essential for community cohesion [80]. For these efforts to be effective, change must begin at the family level, with parents, especially fathers as the heads of the family, taking a more active role in their daughters’ lives [55]. Participants have reported that some men don’t fully understand the extent of the dangers FGM poses in their daughters’ lives until they are properly educated about it, and that education helps protect their daughters from enduring such a challenging life in the future. By addressing the perspectives of all stakeholders, these initiatives can create a supportive environment for rejecting FGM [62].

Despite these successes, the study highlighted challenges in ensuring consistent knowledge transfer, particularly in rural areas. Many communities lack access to schools or structured awareness programs, leaving adolescents reliant on family elders for guidance. This challenge is particularly pronounced in low- and middle-income countries, where socioeconomic factors, geographical disparities, and cultural norms create significant barriers to structured health education [39, 81]. In such cases, traditional beliefs often dominate, perpetuating the cycle of FGM. This disparity underscores the need to expand educational initiatives to underserved areas, ensuring that all young people, regardless of location, have access to accurate information about FGM. Formal education has proven to be a key factor in reducing the prevalence of FGM among girls [13, 17]. However, the impact of education on completely eliminating the practice may take considerable time to manifest [48].

Overall, at the individual level, participants indicated enhanced awareness and a repudiation of myths associated with FGM with chastity, honour, and moral authority. However, stated intentions to refrain from circumcising daughters were frequently contingent on acceptance by the wider family and community, suggesting that cognitive transformation does not inherently result in behavioural abandonment. Interpersonally, the decision to have FGM was still made within the dynamics of marriage and extended family. Even though the intervention made it easier for spouses to talk to each other, generational hierarchies, especially the influence of older women, still affected the results. This emphasises that FGM remains a socially coordinated practice rather than an individual decision.

At the community level, new counter-narratives point to slow changes in norms, especially among younger people. However, older generations’ symbolic attachment to tradition shows that change is not happening evenly and that there could be cultural tension. It was found that organisational actors, especially religious and community leaders, were particularly important for making anti-FGM messages more credible. But the lack of emphasis on policy enforcement shows a gap between the legal framework and the lived experience of people in the community.

The SEM shows that sustainable abandonment requires more than just awareness; it requires a multi-level constructive collaboration that addresses social norms, power structures, and institutional reinforcement simultaneously.

## Limitations

The research was limited to participants in Ibadan, Nigeria, which may restrict the generalizability of the findings to other regions or countries with differing cultural and social dynamics. As the data were self-reported, there is a possibility of participants underreporting or exaggerating their responses due to social desirability or sensitivity surrounding FGM. While qualitative data provide depth, the absence of complementary quantitative data limits the ability to measure the statistical significance of the findings or trends.

Additionally, owing to the distance between the researcher and participants, the ethnographic research method, which focuses exclusively on the cultural perspectives of people in a setting, could not be used in this study because the first author, who collected the data, was not physically present. He was in the United Kingdom as a full-time international student and did not have the permission, time, or resources to travel to Nigeria for face-to-face participant recruitment or data collection. Given this dilemma and the strict deadline to complete the project, the most suitable method for recruitment and data collection was an online approach. This meant that each participant should have a smartphone or computer device and be able to use MS Teams. The educated, technologically savvy group became the group that could realistically participate in this study. The study participants were judged adequate to answer the research question.

Being physically present to recruit participants and collect data would have added to the richness of the research findings by allowing further observations of participants over time and by taking field notes of what was observed alongside interviews.

## Conclusion

This study highlights the critical role of awareness programs in shaping perceptions and promoting behaviour change regarding Female Genital Mutilation (FGM) in Ibadan, Oyo State. While the findings suggest that these programs have made measurable progress in reducing the prevalence of FGM, especially in urban settings, deeply entrenched cultural norms and generational divides remain formidable barriers. The narratives of the participants reveal that culturally sensitive, community-driven education initiatives—particularly those integrated within schools, health centers, and religious institutions—are effective in challenging traditional beliefs and empowering young adults to reject harmful practices.

However, resistance from older generations and limited access to structured awareness platforms in rural areas continue to impede broader social transformation. Bridging these gaps will require expanded outreach efforts, active engagement with community gatekeepers, and the inclusion of intergenerational dialogue within intervention frameworks. A focus on grassroots volunteerism and partnerships with local leaders may further strengthen public health messaging and community buying. Future studies can contribute to a deeper understanding of FGM beliefs in rural areas, especially among older adults.

## Supplementary Information

Supplementary Material 1

## Data Availability

The datasets generated and analysed during the current study are not publicly available due to ethical constraints but are available from the corresponding authors on reasonable request.
