# Supplementary material for: Community awareness and cultural beliefs on female genital mutilation in Ibadan, Oyo State: Insights from a localised intervention
Source: BMC Womens Health. 2026 Apr 27;26:295. doi: 10.1186/s12905-026-04483-2 (PMC13267586; doi:10.1186/s12905-026-04483-2)
Supplement: Supplementary file 1 — Supplementary Material 1 [file 12905_2026_4483_MOESM1_ESM.docx]

**Interview Guide**

***Title:*** *Community Awareness and Cultural Beliefs on Female Genital Mutilation in Ibadan, Oyo State: Insights from a Localized Intervention*

**Part 1: Personal Information**

1. Could you please briefly tell me about yourself? (Prompts: What do you do? How long have you lived in Ibadan? Are you originally from Ibadan? What motivated you to take part in this study?

**Part 2: Attitudes and Perceptions Towards FGM**

1. What are your initial thoughts and feelings when you hear the phrase/words Female Genital Mutilation?
2. What can you now tell us about your understanding on Female Genital Mutilation (FGM)?
3. What do you perceive as the main reasons for the practice of FGM in your community?

**Part 3: Impact of Awareness Programs**

1. Have you ever participated in any awareness programs or campaigns about FGM, either in private or in a group? If yes, please can you provide details of the programs you have participated in?
2. What role do you think awareness programs can do in the prevalence of FGM?
3. How do you feel that awareness programs can have any impact to reducing the practice of FGM in your community?
4. Have you witnessed any changes in community attitudes towards FGM because of awareness programs? On a scale of 1-10, can you rate the impact of FGM awareness program on your community
5. What do you think are the most effective strategies for raising awareness about FGM in your community?
6. What other thing do you think awareness programs can sensitize our community about which could have been done to replace FGM in keeping our ladies chaste instead of the practice FGM?
7. What is now your opinion about the practice of FGM that you may likely pass on to upcoming generations?
8. What other support do you think a victim of FGM should receive to complement their level of awareness?
9. Is there anything else you would like to share about your experiences or thoughts regarding FGM and awareness programs in your community?

Thank you for participating in this survey. Your input is valuable in helping us understand the impact of awareness programs on FGM in Ibadan, Oyo State, Nigeria.
